# Insight into chloroplast genome structural variation of the Mongolian endemic species* Adonis mongolica* (Ranunculaceae) in the Adonideae tribe

**DOI:** 10.1038/s41598-023-49381-x

**Published:** 2023-12-12

**Authors:** Nudkhuu Nyamgerel, Shukherdorj Baasanmunkh, Batlai Oyuntsetseg, Gun-Aajav Bayarmaa, Andrey Erst, Inkyu Park, Hyeok Jae Choi

**Affiliations:** 1 Department of Biology and Chemistry, Changwon National University, Changwon, 51140 South Korea; 2 Department of Biology, School of Arts and Science, National University of Mongolia, Ulaanbaatar, 14201 Mongolia; 3 Central Siberian Botanical Garden, Siberian Branch of the Russian Academy of Science, Novosibirsk, 630090 Russia

**Keywords:** Plant sciences, Biological techniques, Genomic analysis

## Abstract

*Adonis mongolica* is a threatened species that is endemic to Mongolia. It is a medicinal plant from the *Adonis* genus and has been used to treat heart diseases. However, the genomics and evolution of this species have not been thoroughly studied. We sequenced the first complete plastome of *A. mongolica* and compared it with ten Adonideae species to describe the plastome structure and infer phylogenetic relationships. The complete plastome of *A. mongolica* was 157,521 bp long and had a typical quadripartite structure with numerous divergent regions. The plastomes of Adonideae had relatively constant genome structures and sizes, except for those of *Adonis*. The plastome structure was consistent across *Adonis*. We identified a 44.8 kb large-scale inversion within the large single-copy region and *rpl*32 gene loss in the *Adonis* plastomes compared to other members of the Adonideae tribe. Additionally, *Adonis* had a smaller plastome size (156,917–157,603 bp) than the other genera within the tribe (159,666–160,940 bp), which was attributed to deletions of intergenic regions and partial and complete gene losses. These results suggested that an intramolecular mutation occurred in the ancestor of the *Adonis* genus. Based on the phylogenetic results, *Adonis* separated earlier than the other genera within the Adonideae tribe. The genome structures and divergences of specific regions in the *Adonis* genus were unique to the Adonideae tribe. This study provides fundamental knowledge for further genomic research in Mongolia and a better understanding of the evolutionary history of endemic plants.

## Introduction

*Adonis* L., comprising 26–38 species, belongs to the Ranunculaceae family^1–3^. The higher classification of *Adonis* is the Adonideae tribe^4,5^. This genus is commonly distributed in southwestern Asia, Europe, Northern Africa, and the Mediterranean region^1,6^. The general morphology of *Adonis* is characterized by therophytes or perennials, compound leaves, 1–2 palmately or 2–3 pinnately dissected terminal inflorescences, and white, red, and yellow flowers^6,7^. The base number of chromosomes in the genus is x = 8. In perennial species, the somatic number of chromosomes is 2n = 16, and polyploid races with 2n = 24 and 2n = 32 have been identified^8,9^. *Adonis* is divided into two subgenera, *Adonanthe* and *Adonis*, which are perennial and annual species, respectively.

**Figure 1:**
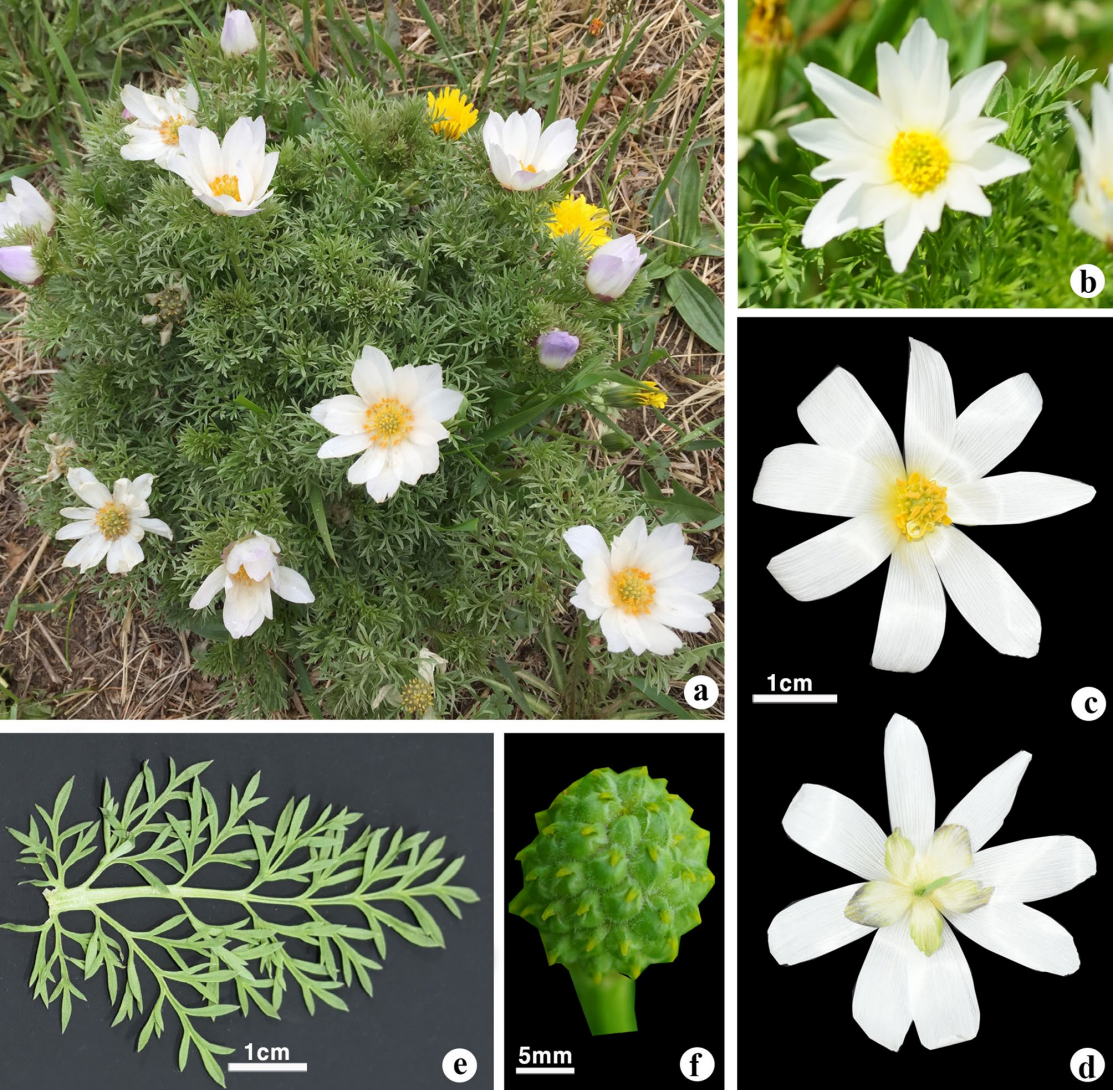
Photographs of wild *Adonis mongolica* of (**a**) general habitat, (**b**) mature flower, natural habitat, (**c**) flower, front view, (**d**) flower, dorsal view, (**e**) leaves, (**f**) aggregated fruit. Photographs by B. Oyuntsetseg (**a**) and Ch. Javzandolgor (**b**–**f**).

*Adonis mongolica* Simonovich is an important medicinal plant that is endemic to Mongolia^10,11^. Since 2000, it has been identified in only two locations in the country, which is of great concern^12^. The general morphology of *A. mongolica* is a plant height of 10–20 cm, with numerous white flowers and violet sepals (Fig. 1). *A. mongolica* inhabits a specific environment compared with other *Adonis*; however, previous studies on *A. mongolica* have primarily focused on its chemical components and medical usage^13–15^, in addition to its distribution and conservation^12,16^. However, the genetic background of *A. mongolica*, along with its phylogenetic relationships and classification, has not been determined. Similar to other *Adonis* species, *A. mongolica* has been used to treat heart failure with symptoms of tachycardia and edema^13^. Currently, *A. mongolica* is critically endangered globally because of overexploitation and loss of habitat^16^. Therefore, implementing programs for protecting and genetic breeding of threatened species is critical. Most *Adonis* species grow where topographical conditions allow for a water supply^17^, but *A. volgensis*, *A. villosa*, and *A. mongolica* grow mostly on drier montane and forb steppes, and these species show evidence of ecological vicariance^18–21^. The evolution of *A. mongolica* was most likely accompanied by the alteration and extension of its ecological constitution.

Over the past few decades, several researchers have studied the whole chloroplast genome (plastome) sequences of plants worldwide. This is because using the plastome sequences to resolve relationships between vascular plants at higher taxonomic levels is more successful than using short-sequence fragments^22,23^. Furthermore, in most angiosperm species, plastome inheritance is uniparental and highly conserved and provides important phylogenetic data^24^. The first detailed plastome study of *Adonis* in 1998 described the restriction site maps for the two species^25^. Currently, the plastomes of four *Adonis* species are available in the NCBI GenBank database^26–28^, accounting for only 2% of the total species diversity. According to previous plastome studies, the plastomes of the genus *Adonis* have large inversions and gene transpositions^25,27^, and the genus belongs to the Adonideae tribe along with *Calathodes, Megaleranthis*, and *Trollius*^26–28^. The Adonideae tribe was derived from its ancestor around 25.5 Mya^28^. In addition, phylogenetic relationships within the *Adonis* genus have been evaluated using partial barcoding genetic markers such as nuclear internal transcribed spacers (ITS)^6,29–31^, *trn*L-F^29^, and random amplified polymorphic DNA (RAPD) markers^30^. However, these studies mostly used several species in sect. *Adonanthe*, and the entire *Adonis* genus has not been studied. Therefore, our study provides fundamental phylogenomic data for *Adonis* and lays the foundation for future phylogenetic studies of Adonideae.

This study aimed to characterize the plastome of *A. mongolica* and identify genetically variable regions through comparison with closely related species within the Adonideae tribe. The plastome results provide useful information for future studies, such as evolutionary analyses and safe medical applications of *A. mongolica*.

## Results

### Assembly and characteristics of the *A. mongolica* plastome

**Figure 2:**
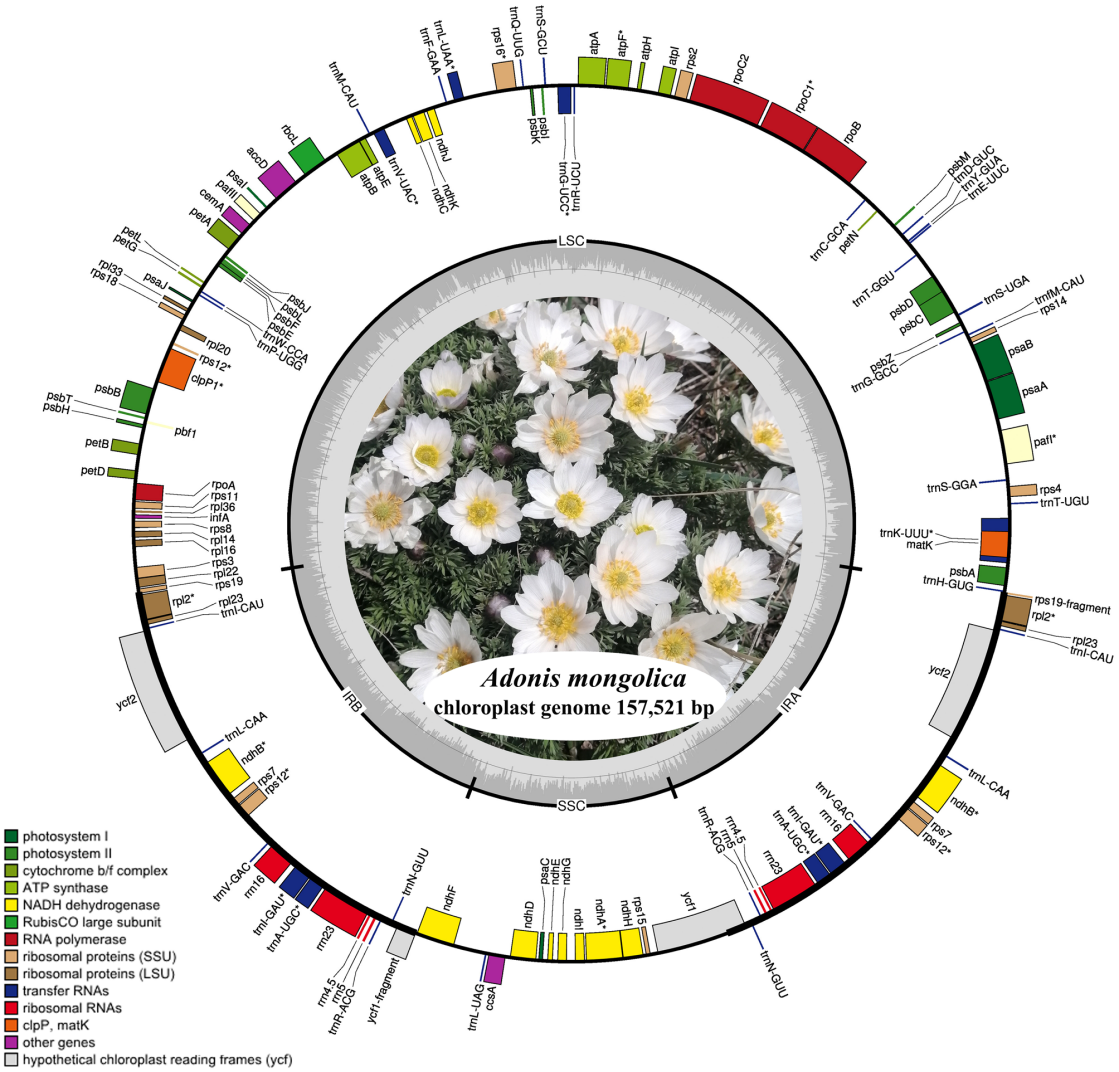
Circular gene map of the complete chloroplast genome of *Adonis mongolica*. Genes drawn inside the circle are transcribed clockwise, and those outside, counterclockwise. The darker gray in the inner circle represents GC content and light gray represents AT content. Intron containing genes are marked with a star.

The plastome of *A. mongolica* was sequenced for the first time. A total of 8 Gb paired-end (150 bp) clean reads were generated. After trimming and normalization, a list of 22,993,276 paired reads was obtained for de novo assembly (Table S1). De novo assembly generated a single contig, 157,521 bp in length. The plastome of *A. mongolica* contained a large single-copy (LSC; 86,651 bp), a pair of inverted repeats (IR; 26,332 bp), and a small single-copy (SSC; 18,206 bp). The total GC content in *A. mongolica* was 37.9%. Particularly, the GC contents in the IR, LSC, and SSC regions were 43, 36.1, and 31.4%, respectively (Table 1), indicating that the IR region had a higher GC content than the SSC and LSC regions. *Adonis mongolica* contained 113 unique genes. The numbers of rRNA, tRNA, and protein-coding genes in the plastome were 4, 30, and 79, respectively (Fig. 2 and Table S2). Overall, the protein-coding sequences (CDS) were 78,470 bp long, whereas the non-coding sequences (including the intergenic spacer [IGS] and introns) were 79,051 bp long.

**Table 1:** Basic plastomes information of five *Adonis* species.

Species		*A. mongolica*	*A. amurensis*	*A. pseudoamurensis*	*A. coerulea*	*A. sutchuenensis*
Nucleotide length (bp)	Total	157,521	157,032	156,917	157,033	157,603
	LCS	86,651	86,218	86,262	86,545	86,624
	IR	26,332	26,301	26,294	26,087	26,355
	SSC	18,206	18,212	18,067	18,314	18,269
GC content	Total	37.9	38	37.9	37.9	37.8
	LCS	36.1	36.2	36.1	36.1	36.1
	IR	43	43.1	43.1	43.1	43
	SSC	31.4	31.5	31.6	31.3	31.4
Number of the gene	Total	130	130	130	130	130
	CDS	79	79	79	79	79
	tRNA	30	30	30	30	30
	rRNA	4	4	4	4	4
Accession numbers		OQ569932	MW042677	MZ197990	MK253469	MK569470

### Organization of plastomes of the Adonideae tribe

The plastome sequences of 11 Adonideae species (*A. mongolica*, *A. amurensis* Regel & Radde, *A. pseudoamurensis* W.T.Wang, *A. sutchuenensis* Franch., *A. coerulea* Maxim., *Calathodes oxycarpa* Sprague, *Megaleranthis saniculifolia* Ohwi, *Trollius macropetalus* (Regel) F. Schmidt ex W.T.Wang, *T. chinensis* Bunge, *T. farreri* Stapf, and *T. ranunculoides* (Sm.) Steam) were analyzed in this study (Table S3). The complete circular plastomes were 156,917–160,191 bp in length and included an LSC of 86,218–88,944 bp, an SSC of 18,067–18,532 bp, and a pair of IR regions of 26,087–26,632 bp (Table 1 and Table S4). The overall GC content was 37.9–38.1% and was slightly higher in the IR than in the LSC and SSC regions. The Adonideae plastomes contained 131 genes, including 80 protein-coding, 30 tRNA, and 4 ribosomal RNA genes (Fig. S1). Six protein-coding, seven tRNA, and four rRNA genes were duplicated within the IR regions. The complete plastome harbored 18 intron-containing genes, 16 of which had a single intron and two of which had two introns (Tables S5 and S6). Three intron-containing genes were found to be duplicated in the IR regions. The *rps*12 gene was a trans-spliced gene with the 5′-end located in the LSC and the 3′-end located in an IR region.

Analysis of codon usage and anticodon recognition patterns revealed 26,210–26,427 codons, of which leucine, serine, and isoleucine were the most abundant (Fig. S2). To identify codon patterns, we analyzed codon distribution in 11 plastomes (Fig. S3). Codons with A or T at the third position had a strong codon bias. Most Adonideae displayed a comparable pattern with high RSCU values for arginine (AGA), leucine (TTA), and alanine (GCT).

### Repeat sequences

**Figure 3:**
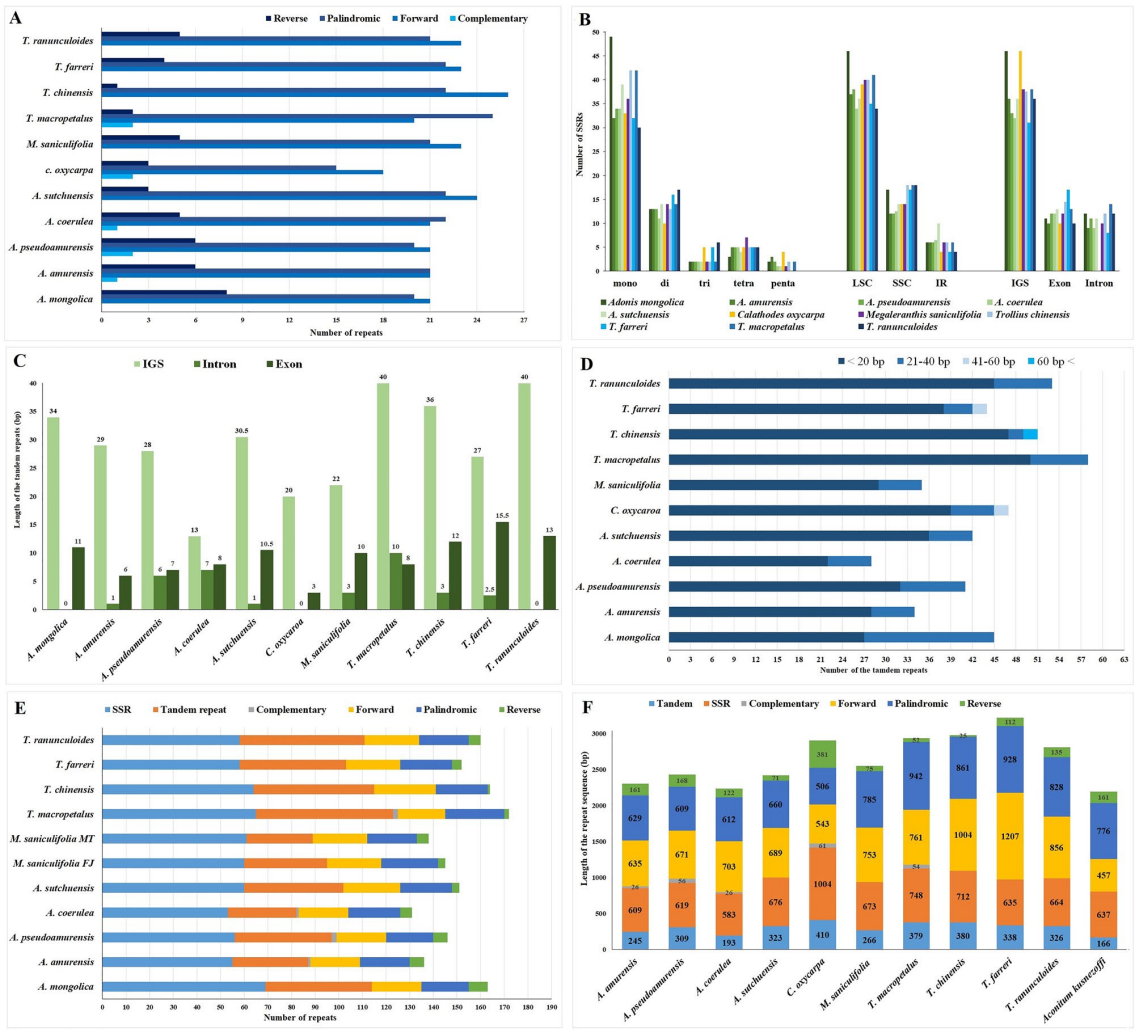
The distribution of repeat sequences in of tribe Adonideae plastomes. (**a**) The number of repeat types in Adonideae plastome. (**b)** The number of simple sequence repeats (SSR) motifs and the distribution of SSRs in the genome regions. (**c**) Distribution of tandem repeats in intergenic region (IGS), intron, and exon. (**d**) Total number of tandem repeats. (**e**) Distribution of repeats in genomic regions. (**f**) Percentage of repeats in genomic regions.

We investigated the repeat sequences of Adonideae species to determine their characteristics and proportions of repeat sequences within the tribe. Adonideae species had a similar number of forward, reverse, palindromic, and complementary repeats (Fig. 3a). For single-sequence repeats (SSRs), mononucleotide motifs were the most abundant in all species, followed by dinucleotide motif repeats (Fig. 3b). A total of 53–69 SSRs were identified, mostly in the LSC region and particularly within the IGS region. Most of the tandem repeats were located in the IGS region (Fig. 3c). We found 23–58 tandem repeats that were generally 9–68 bp long (Fig. 3d). Among the tandem repeats, three regions (*rps*16-*ndh*J, *ndh*F-*ccs*A, *ndh*G-*ndh*I) in the IGS and the *ycf*2 gene in the LSC region were common among the five *Adonis* plastomes. The number of repeat sequences was the highest in *T. macropetalus* (Fig. 3e). However, the tandem repeats were shorter than the other repeat sequences, and this pattern was similar in all the Adonideae examined (Fig. 3f).

### Plastome comparison

**Figure 4:**
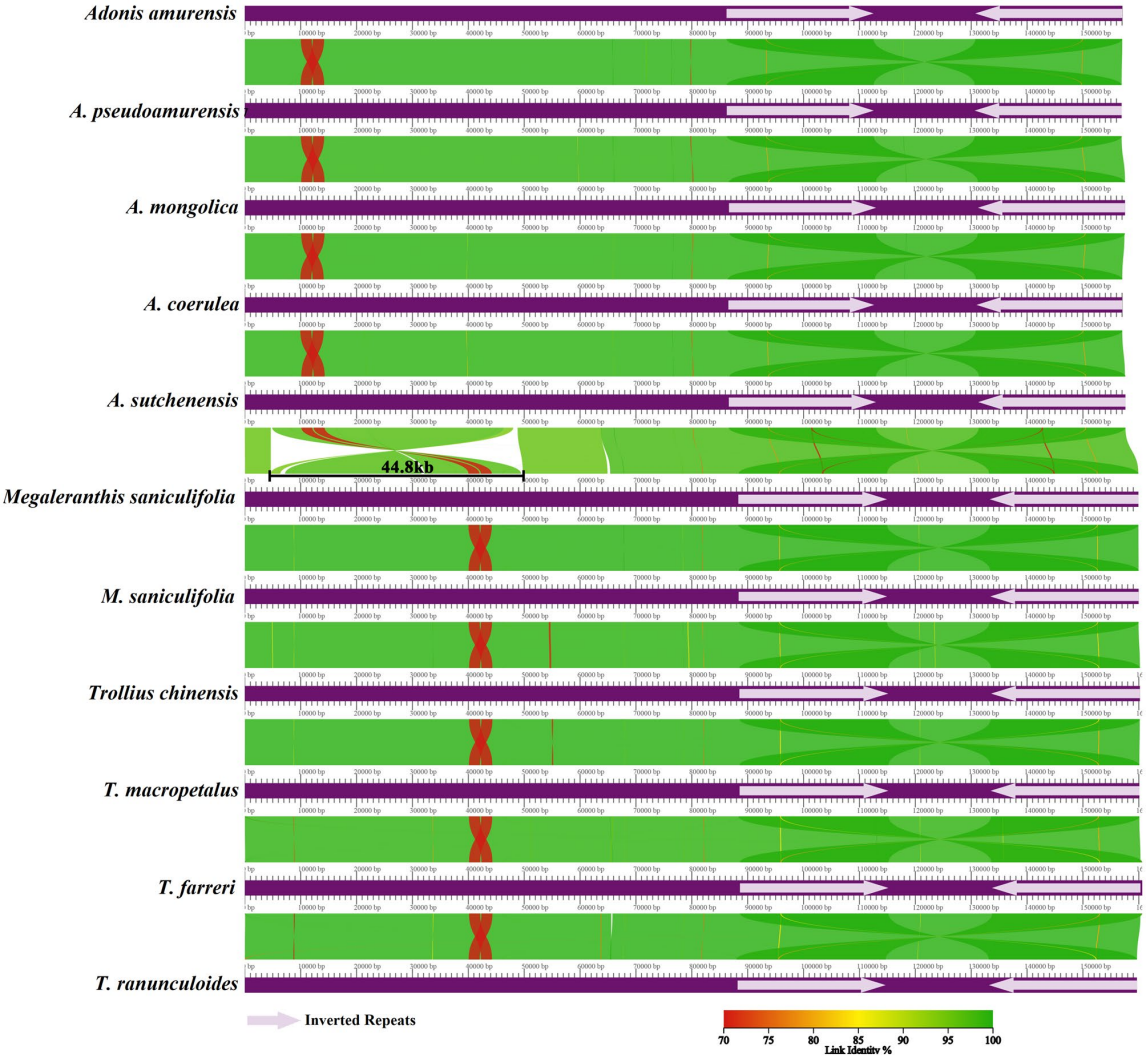
The complete plastome structure of Adonideae species. Linear maps were built using the AliTV visualization software, based on whole genome alignment. Both panels depict pairwise comparisons, expressed as percentage of nucleotide similarity, that connect different homologous genomic regions. Genomes are completed in full and pictured in purple.

The genome structure, gene order, and boundaries between IRs and single-copy regions were similar among Adonideae plastomes; however, genus-specific rearrangements in *Adonis* plastomes were revealed. Collinearity in gene placement among the 11 available Adonideae plastomes was assessed using AliTV (Fig. 4 and Fig. S4). From the *trn*T-UGU to the *rps*16 genes, a 35-gene inversion (21 protein-coding genes and 14 tRNAs), 44.8 kb in length, evolved in *Adonis*. Furthermore, two deletion sites (844 and 524 bp) in the intergenic regions were found within the inversion region of the *Adonis* plastomes. This inversion was not detected in other available species within the tribe. In Adonideae, IR lengths ranged from 26,087 to 26,632 bp, but the borders between the IR regions and the two single-copy regions (LSC and SSC) were similar. The *rps*19 and *ycf*1 genes spanned the LSC/IRb and SSC/IRa junctions, respectively (Fig. S5). Only IR contraction was observed in *A. coerulea* because the *rps*19 gene was located entirely in the LSC region.

**Figure 5:**
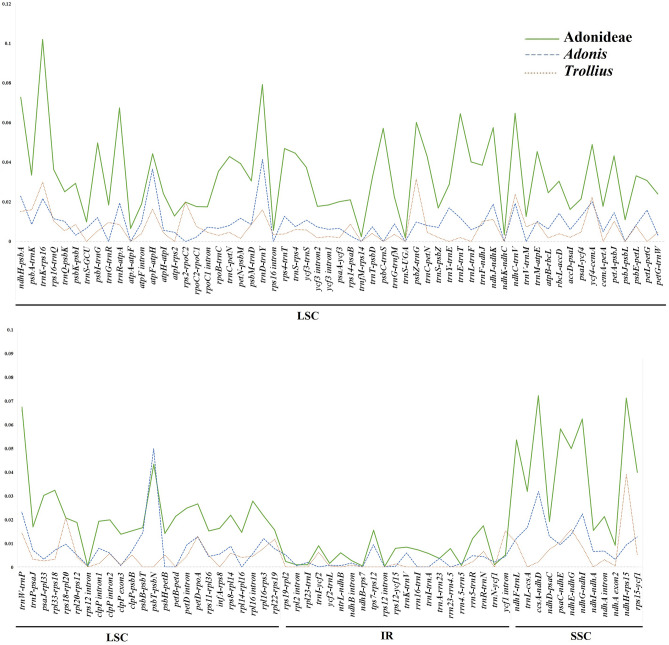
Comparison of the nucleotide diversity (Pi) values of intergenic regions among Adonideae. The mean Pi value of intergenic regions within the tribe Adonideae and the genera *Adonis* and *Trollius* is indicated by green, blue and orange lines, respectively.

The sequence identities of the 11 Adonideae were analyzed using mVISTA, with the *A. mongolica* plastome serving as a reference. We replaced the inversion of *Adonis* to calculate the sequence identity of each gene. As expected, the genic regions were more conserved than the intergenic regions when comparing the ten species (Fig. S6). This pair of IR regions was highly conserved, followed by the LSC and SSC regions. We analyzed the genetic divergence of genes and intergenic regions within Adonideae plastomes, and genus-related mutations were detected using pairwise comparisons. Overall, Adonideae plastomes had an average genetic diversity (Pi) value of 0.018. The highest Pi values of the IGS regions were observed for *trn*K*-rps*16 (0.102) in SSC (Fig. 5). The *ycf*1, *rps*16, *ndh*F, and *mat*K genes are variable within the Adonideae species. Species-specific mutations of *Adonis* were detected using pairwise alignment. The highest Pi value was detected for *psb*T-*psb*N (0.104) in *A. mongolica* and *A. coerulea* (Fig. S7). In addition, the *ndh*F-*trn*L IGS showed considerable length variation in Adonideae because the *rpl*32 gene was completely lost in *Adonis* (Fig. S8).

### Phylogenetic analysis

**Figure 6:**
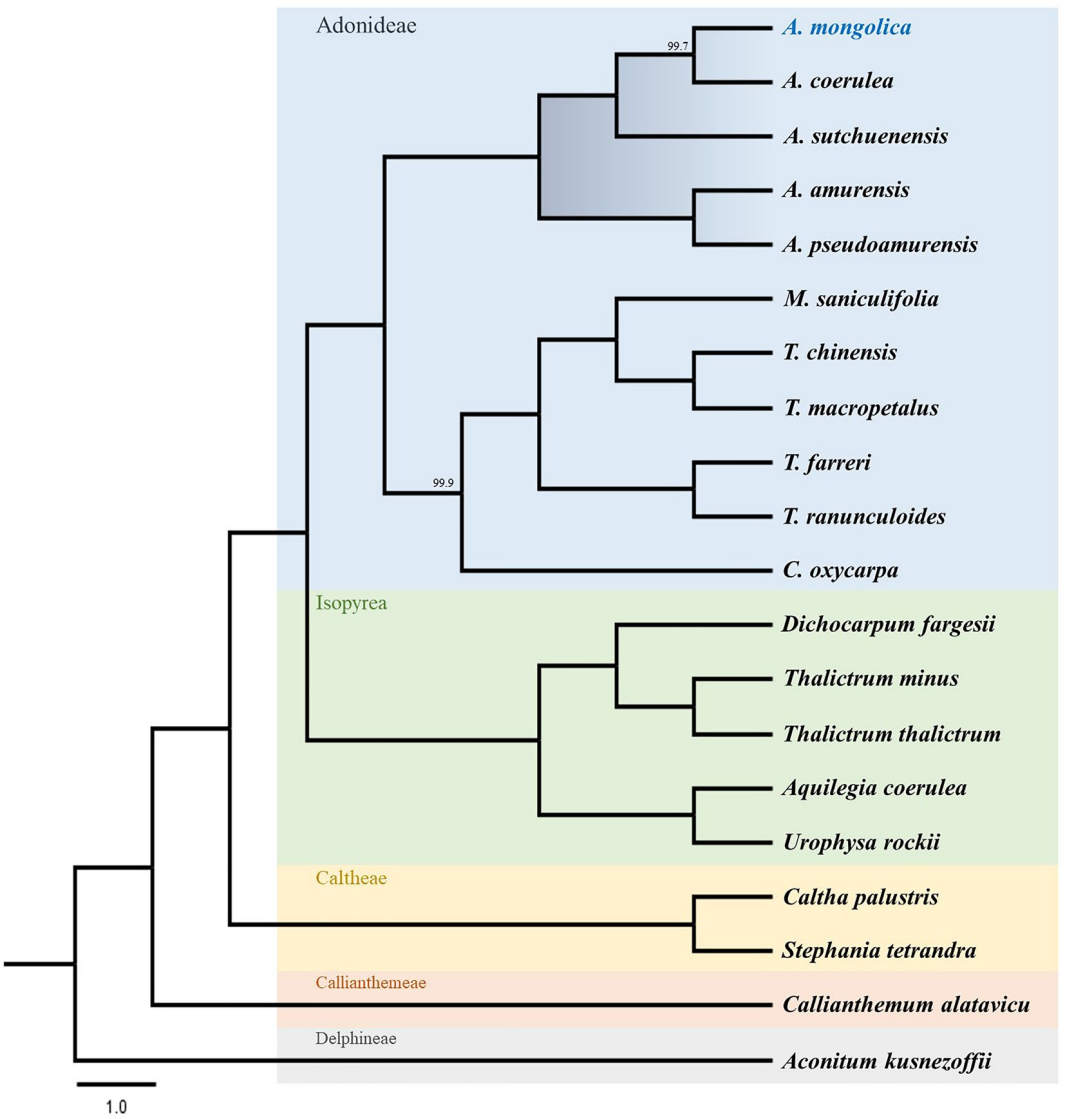
Phylogenetic tree from Ranunculaceae species based on the whole plastomes using Maximum parsimony (MP), Bayesian inference (BI), and Maximum likelihood (ML). The MP topology is indicated with BI probabilities and ML bootstraps at each branch; maximum support values are not indicated (MP 100, PP 1, and ML 100). The plastome completed in this study is indicated with bold font.

Two datasets were compiled to verify the phylogenetic relationships among members of the Adonideae tribe. The first dataset consisted of 80 conserved protein-coding sequences of 11 Adonideae species, obtained by aligning 87,075 bp, of which 82,024 bp (94.2%) were constant and 5051 bp (5.8%) were parsimony-informative. The second dataset contained the complete plastomes of 17 Ranunculaceae species. Due to the relatively high variation within the intronic and intergenic regions, the matrix contained 200,666 nucleotide sites, of which 152,797 bp (76.1%) were constant and 37,887 bp (18.9%) were parsimony-informative among Ranunculaceae. The *Aconitum kusnezoffii* Rchb. plastome served as the outgroup for both datasets. The *Adonis* species were distinct from other genera in the Adonideae tribe and were supported by strong bootstrap values and posterior probabilities (PPs) for the CDS and whole plastome datasets (Fig. 6 and Fig. S9). *Adonis mongolica* clustered with *A. coerulea* and *A. sutchuenensis*, whereas *A. amurensis* and *A. pseudoamurensis* were positioned as sister taxa in this cluster. However, the relationship between *A. mongolica*, *A. coerulea*, and *A. sutchuenensis* was differently positioned in the two datasets: in the whole plastome dataset, *A. mongolica* and *A. coerulea* were more closely related than *A. sutchuenensis* (Fig. 6); while, in the CDSs dataset, *A. coerulea* and *A. sutchuenensis* were more closely related than *A. mongolica* (Fig. S9).

## Discussion

Plastomes have been widely used as models to elucidate patterns of genetic variation in space and time, as well as micro- and macro-evolutionary events across all plant lineages^32^. This is because the plastome is highly conserved, with evolutionary hotspots, such as gene insertions and deletions, IR contractions and expansions, inversions, and various repeat sequences^33^. We sequenced and characterized the plastome of *A. mongolica* and conducted comparative studies that included ten other species of the Adonideae tribe to identify genetic variations that could explain the evolutionary changes. The complete plastome of *A. mongolica* showed a typical quadripartite structure with one LSC, one SSC, and two IR regions, which was a highly conserved pattern. However, unique structural rearrangements were detected in the newly sequenced *A. mongolica*, as with other species of *Adonis*. Multiple inversions and transpositions have been detected in the LSC regions of Ranunculaceae plastomes^27,34^. The *Adonis* genus plastome includes a 44.8 kb inversion in the LSC region^25,27,28^. Inversions arise when two breaks occur within a DNA segment, and the segment between the two breaks inserts itself in the opposite direction, causing a 180° inversion relative to the adjacent region. Inversion breakpoints are frequently found in regions with repetitive nucleotides, and these regions may be reused in future inversions^35^. Repeated sequences have been discovered at the endpoints of several land plant plastome inversions, as well as in tRNA genes^36^. In the *Adonis* genus, the inversion reversed the order of genes between *rps*16 and *trn*T-UGU, placing *trn*K-UUU adjacent to *trn*T-UGU and *rps*16 adjacent to *trn*L-UAA. This inversion occurred between two tRNA genes; however, there was no repeat region near the endpoint of the inversion. This might have been caused by random changes in the *Adonis* plastome. Furthermore, large-scale inversion occurred in the ancestor of *Adonis* after being derived from the Adonideae tribe because this type of inversion was not observed in any other genus within the tribe. Plastome sequences have been used by many researchers to determine phylogenetic relationships at various taxonomic levels^37^. Early molecular phylogenetic studies have revealed that *Adonis* is related to *Trollius*^38^. Based on floral development and phyllotaxis, *Adonis* was assigned to the Adonideae tribe and is closely related to the genus *Calathodes*^5^. In addition, the phylogenetic relationship of Ranunculaceae was revised using plastome analysis, revealing that *Adonis* belonged to the Adonideae tribe^27,28,39^. Our phylogenetic study revealed the same topology as previous studies; the Adonideae tribe formed a monophyletic clade, which includes *Adonis*, *Calathodes*, *Megaleranthis*, and *Trollius* based on whole plastome and protein-coding genes. Within this tribe, the *Adonis* genus formed a distinct monophyletic group (Fig. 6).

The plastome size of the Adonideae species investigated here varied slightly, with that of the *Adonis* genus being shorter (156,917–157,603 bp) than those of the other Adonideae species (159,666–160,940 bp). *Adonis* plastome size diversity was detected in LSC and IR. The IR contraction occurred due to a partial deletion (200 bp) of the *ycf*2 gene, reduction of the LSC region due to a ~ 2 kb nucleotide loss during large-scale inversion, and intergenic deletions in *rps*16-*trn*Q-UUG and *psb*M-*trn*D-GUC regions. The *ycf*2 gene is the largest plastid gene reported in angiosperms. The function of *ycf*2 is largely unknown; however, it does not appear to be related to photosynthesis^40^. The *ycf*2 gene within Ranunculaceae species shows genetic divergence with many repeat events and deletions^41,42^. The *ycf*2 gene could be an alternative to comprehensive genes for studying the relationships between Ranunculaceae species. More than 69 SSRs were identified in *A. mongolica*, and most repeats were in the *ycf*2 gene. This provides a rare opportunity to study the population genetics of this threatened plant, which is endemic to Mongolia. In addition, length variation was detected in the *ndh*F-*trn*L intergenic region within Adonideae. This spacer includes the gene *rpl*32, which produces the ribosomal protein L32^43^; however, the gene has been completely deleted in the *Adonis* genus^27,34,39,44^. This spacer within the Ranunculaceae plastome is either a pseudogene or absent and has a wide range of lengths with a 1.6 to 5.5-fold reduction compared to that of the full-length IGS of *rpl*32^45^. Due to various events of complete loss, this IGS region in *Adonis* is nearly 0.8 times shorter than that in other Adonideae genera. Transfer of *rpl*32 from the plastome to the nucleus has been observed in Euphorbiaceae^46^, Ranunculaceae^27,45^, Rhizophoraceae^47^, and Salicaceae^48^; thus, the *rpl*32 may have been transferred to the nucleus or subjected to complete loss in the *Adonis* plastome. Furthermore, some coding regions had significantly high diversity, such as the *ycf*1, *rps*16, and *mat*K genes (Fig. 5). These highly variable regions may be useful as specific DNA barcodes for species-level identification, as well as for providing genetic markers for resolving relationships among the Adonideae.

Taxonomic studies of the *Adonis* genus have not been well revised; the species of sect. *Adonanthe* were mostly studied, including the nuclear ITS region^6,29,30^ and the *trn*L-F^29^, *atp*B, and *rbc*L^38^ regions of the plastome. In the present study, we estimated the genetic diversity of the plastomes of five species of sect. *Adonanthe*. The *ccs*A-*ndh*D, *ndh*D-*ndh*I, and *trn*D-*trn*Y regions showed different haplotypes for each species (Fig. S7). These regions can be used to identify species within the *Adonis* genus. Among the modern barcoding markers, *trn*H-*psb*A (0.023) was highly variable for *Adonis*, whereas the previously used *trn*L-F (0.006), *atp*B (0.005), and *rbc*L (0.004) markers were relatively less variable. Based on our observations, partial genetic markers of the plastome are unsuitable for identifying species within the *Adonis* genus. Based on whole plastome sequences, *A. mongolica* clustered with *A. coerulea* and belonged to subg. *Adonanthe* sect. *Adonanthe* ser. *Coerulea*. The results revealed the morphological characteristics of petiolate leaves, rhizomes, trichomes on achenes, and white or purple petals^49^. The species of ser. *Coerulea* has numerous mixed light-purple and white flowers, whereas *A. mongolica* and *A. davidii* have only white flowers^7,18^. Interestingly, *A. mongolica* was a sister taxon to the cluster containing *A. coerulea* and *A. sutchuenensis* based on protein-coding genes. Specific mutations may have occurred in protein-coding genes of *A. mongolica*. Many studies have revealed that mutations in genes corresponding to flower color can affect adaptive success^50,51^. Future studies on the gene expression of flower color will be valuable for a better understanding of the adaptation and evolution of the *Adonis* genus.

The results of the present study expand the genetic information on medicinal plants endemic to Mongolia. In particular, *A. mongolica* has numerous genetic divergence regions; *Adonis* members show a genus-specific 44.8 kb inversion, several partial deletions, and complete gene loss. These changes have not been detected in other genera within the tribe; thus, they occurred in the ancestor of *Adonis* after its separation from the Adonideae tribe. This may have been caused by nonrandom recombination associated with climate change, making it an interesting topic for future evolutionary investigation.

## Conclusions

The plastome of *A. mongolica* was sequenced and annotated for the first time. It included a highly conserved gene order, identical to that of the *Adonis* species, at 157 kb, and has a median plastome size for photosynthetic land plants. We identified important genetic resources for sequence divergence and phylogenetic inference of *A. mongolica*, providing a rare opportunity to study the genetic background of this threatened plant endemic to Mongolia. Comparative genomics indicated that *Adonis* plastomes were relatively conserved but included several genus-specific rearrangements and highly divergent locations. For instance, we identified a uniquely large inversion in the LSC region, complete gene loss from the SSC region, and some genes and intergenic regions with high sequence diversity. This study provides valuable genetic information to understand the evolutionary relationships of the genus *Adonis* within the Adonideae tribe.

## Materials and methods

### Plant collection, DNA sequencing, and plastome assembly

The plant material used in this study was collected from natural populations in Argalant Soum, Tuv province (N47.887992 and E106.41658), Mongolia, and a voucher specimen (UBU0032866) was deposited in the herbarium of the National University of Mongolia, Mongolia. Total DNA was extracted from the tested species by using a modified CTAB method^52^. The library was prepared from total genomic DNA using the TruSeq DNA Nano Kit on the NextSeq 500 platform (Illumina, San Diego, CA, USA), following the manufacturer’s protocol. Trimmomatic v. 0.36^53^ was used to remove adapter sequences and low-quality reads to reduce biases in the analysis. The total number of bases, reads, GC (%), Q20 (%), and Q30 (%) for *A. mongolica* samples were calculated after filtering. A base quality plot generated using FastQC v. 0.11.5^54^ was used to evaluate the overall quality of the data. This plot shows the range of quality values for each cycle. De novo assembly was performed with various k-mers using NOVOplasty^55^. The best k-mer was selected based on the assembly results, including the number of contigs, total contig length, and N50. Read sequences produced using the Illumina platform for plastome assembly were mapped to validate the assembly results, and the depth, coverage, and insert size were calculated. The raw data reads were mapped to an assembly result to identify the insert size of the raw data and the number of reads used in the assembly. The appropriate statistics were calculated after mapping. After the complete or draft plastome was assembled, BLAST analysis was performed to identify the species with which each contig (or scaffold) showed similarity.

### Repeat analysis

We used REPuter to identify forward, reverse, palindromic, and complementary repeats with a minimum length of 20 bp, 90% identity, and a Hamming distance of 3^56^. SSRs were detected using MISA^57^ with the minimum number of repeat parameters set to 10, 5, 4, 3, 3, and 3 for mono-, di-, tri-, tetra-, penta-, and hexanucleotides, respectively. Tandem repeats ≥ 20 bp were identified using the Tandem repeats finder^58^ with a minimum alignment score of 50 and a maximum period size of 500; the identity of repeats was set to ≥ 90%.

### Plastome annotation and comparative analysis

Plastome annotation of *A. mongolica* was performed using GeSeq^59^ to predict the location, whereas BLAST^60^ was used to determine the function of and identify the assembled sequences against nucleotide and protein sequence databases. The complete plastome sequence of *A. mongolica* has been submitted to GenBank (accession number: OQ569932). A total of 11 samples, representing five species from the genus *Adonis*, including *A. mongolica*, four species from the genus *Trollius*, and one species each from the genera *Megaleranthis* and *Calathodes*, were analyzed in this study. OrganellarGenomeDraw^61^ was used to generate circular and linear plastome maps. The GC content and RSCU of the ten plastomes were analyzed using MEGA11 software^62^. The codon usage distribution of Adonideae plastomes was visualized using the Heatmapper tool with average linkage clustering and Euclidean distance measurement methods^63^. An RSCU < 1.00 indicated a codon that was used less frequently than expected, whereas an RSCU > 1.00 indicated a codon that was used more frequently than expected. Pairwise whole plastome alignments of the Adonideae plastomes were visualized using AliTV software^64^ and MAUVE v2.3.1^65^. The SC/IR boundary shifts at four junctions (LSC/IRa, IRa/SSC, SSC/IRb, and IRb/LSC) of the sample plastomes were compared using IRscope^66^. The mVISTA program^67^ in Shuffle-LAGAN mode^68^ was used to detect species-specific genetic variation by comparing Adonideae plastomes with *A. mongolica* as a reference. DnaSP version 6^69^ was used to calculate nucleotide variability (Pi) among plastomes. The CDS, intron, and IGS regions were analyzed separately to confirm the exact genetic variants. Statistical analysis was performed using XLSTAT^70^ to test whether gene loss was related to IGS length.

### Phylogenetic analysis

We constructed two datasets for phylogenetic analysis: CDSs of Adonideae species and complete plastomes of Ranunculaceae species. Complete plastomes and CDSs were individually aligned using MAFFT ver. 7.388^71^. Alignment datasets were filtered to remove ambiguously aligned regions using GBlock ver. 0.91.1^72^. The best-fitting model for nucleotide substitutions was determined using the Akaike information criterion in jModelTest v2.1.10^73^, and the GTR + I + G model was selected for maximum likelihood (ML) analysis. The GTR + G model was selected for the Bayesian inference (BI) analysis. The maximum parsimony (MP) analysis was conducted using PAUP* v4.0b10^74^. The MP searches included 1000 random addition replicates and TBR branch swapping using the MulTree option. ML analysis was performed using RaxML v. 8.0.5^75^ with 1000 bootstrap replicates. BI analysis was carried out using MrBayes 3.2.2^76^, with two independent runs of four simultaneous chains executed for 5,000,000 generations using the Markov chain Monte Carlo algorithm. Trees were sampled every 5000 generations, and the first 25% were discarded as burn-in. The trees were determined using a 50% majority-rule consensus to estimate PPs. Reconstructed trees were visualized using FigTree version 1.4.2^77^.

### Ethics approval and consent to participate

Wild plants were collected and identified as Oyuntsetseg and Baasanmunkh. A voucher specimen was deposited in the herbarium of the National University of Mongolia (UBU) under voucher ID UBU0032866. Permission was obtained from the appropriate governing bodies. This study was conducted in accordance with the relevant guidelines and regulations.

### Supplementary Information

Supplementary Information.

## Data Availability

The complete chloroplast genome of *Adonis mongolica* generated in this study was submitted to the NCBI database (https://www.ncbi.nlm.nih.gov/) with GeneBank accession number OQ569932. All plastomes used in this study can be found in GenBank, and the GenBank accessions are shown in Additional File 1: Table S3. The other chloroplast genomes used in this study were downloaded from the NCBI.
