# Supplementary material for: Insight into chloroplast genome structural variation of the Mongolian endemic species Adonis mongolica (Ranunculaceae) in the Adonideae tribe
Source: Sci Rep. 2023 Dec 12;13:22014. doi: 10.1038/s41598-023-49381-x (PMC10716127; doi:10.1038/s41598-023-49381-x)
Supplement: Supplementary file 1 — Supplementary Information. [file 41598_2023_49381_MOESM1_ESM.docx]

**Supplementary Information**

**Title: Insight into chloroplast genome structural variation of the Mongolian endemic species Adonis mongolica (Ranunculaceae) in the Adonideae tribe**

**Supplementary Table S1** Raw reads and genome assembly information for *A. mongolica* complete chloroplast genome.

| Input reads (bp) | 8,786,984,450 |
| --- | --- |
| Trimmed reads (bp) | 3,438,631,152 |
| Total raw base (bp) | 58,191,950 |
| Trimmed bases (bp) | 22,993,276 |
| Q20 (%) | 88.97 |
| Q30 (%) | 80.31 |
| Aligned reads (#) | 299,097 |
| Coverage (%) | 100 |
| Depth (X) | 260.76 |
| Cp genome length (bp) | 157,521 |

**Supplementary Table S2** Genes in the chloroplast genomes of *Adonis mongolica*.

| Category | Group of genes | Name of genes |
| --- | --- | --- |
| RNA genes | ribosomal RNA genes (rRNA) | *rrn*5, *rrn*4.5, *rrn*16, *rrn*23 |
|  | Transfer RNA genes (tRNA) | *trn*A-UGC, *trn*C-GCA, *trn*D-GUC, *trn*E-UUC, *trn*F-GAA, *trn*fM-CAU, *trn*G-GCC, *trn*G-UCC, *trn*H-GUG, *trn*I-CAU, *trn*I-GAU^a^, *trn*K-UUU^a^, *trn*L-CAA, *trn*L-UAA^a^, *trn*L-UAG, *trn*M-CAU, *trn*N-GUU, *trn*P-UGG, *trn*Q-UUG, *trn*R-ACG, *trn*R-UCU, *trn*S-GCU, *trn*S-CGA^a^, *trn*S-UGA, *trn*T-GGU, *trn*T-UGU, *trn*V-GAC, *trn*V-UAC^a^, *trn*W-CCA, *trn*Y-GUA |
| Ribosomal  proteins | Small subunit of ribosome | *rps*2, *rps*3, *rps*4, *rps*7, *rps*8, *rps*11, *rps*12^c, b, d^, *rps*14, *rps*15, *rps*16^a^, *rps*18, *rps*19 |
| Transcription | Large subunit of ribosome | *rpl*2^a^, *rpl*16^a^, *rpl*14, *rpl*20, *rpl*22, *rpl*23, *rpl*33, *rpl*36 |
|  | RNA polymerase | *rpo*A, *rpo*B, *rpo*C1^a^, *rpo*C2 |
| Protein  genes | Photosystem I | *psa*A, *psa*B, *psa*C, *psa*I, *psa*J, *ycf*3^b^, *ycf*4 |
|  | Photosystem II | *psb*A, *psb*B, *psb*C, *psb*D, *psb*E, *psb*F, *psb*H, *psb*I, *psb*J, *psb*K, *psb*L, *psb*M, *psb*N, *psb*T, *psb*Z |
|  | Cytochrome b6/f | *pet*A, *pet*B^a^, *pet*D^d^, *pet*G, *pet*L, *pet*N |
|  | ATP synthase | *atp*A, *atp*B, *atp*E, *atp*F^a^, *atp*H, *atp*I |
|  | Rubisco | *rbc*L |
|  | NADH dehydrogenase | *ndh*A^a^, *ndh*B^a^, *ndh*C, *ndh*D, *ndh*E, *ndh*F, *ndh*G, *ndh*H, *ndh*I, *ndh*J, *ndh*K |
|  | ATP dependent protease subunit P | *clp*P^b^ |
|  | Chloroplast envelope membrane protein | *cem*A |
| Other genes | Maturase | *mat*K |
|  | Subunit acetyl-coA carboxylase | *acc*D |
|  | C-type cytochrome synthesis | *ccs*A |
|  | Hypothetical proteins | *ycf*1, *ycf*2, *ycf*15^e^ |
|  | Component of TIC complex | *ycf3*^b^ |

^a^ Gene with one intron, ^b^ Gene with two intron, ^c^ Gene with copies, ^d^ Trans-splicing gene, ^e^ pseudogene

**Supplementary Table S3** Species of tribe Adonideae used for comparative chloroplast analysis.

| Tribe | Species | Locality | Accession number |
| --- | --- | --- | --- |
| Adonideae | *Adonis mongolica* | Argalant soum, Tuv province, Mongolia | OQ569932 (this study) |
|  | *A. amurensis* | Benxi, Liaoning, China | MW042677 |
|  | *A. pseudoamurensis* | Benxi, Liaoning, China | MZ197990 |
|  | *A. coerulea* | Xiaojin, Sichuan, China | MK253469 |
|  | *A. sutchuensis* | Taibai Mountain, Baoji, China | MK569470 |
|  | *Megaleranthis saniculifolia* | Sobaek Mountains, Sancheong, Korea | FJ597983 |
|  | *Trollius macropetalus* | Harbin, Heilongjiang, China | MW308598 |
|  | *T. chinensis* | Institute of Botany, CAS, Beijing, China | MK569501 |
|  | *T. farreri* | Taibai Mountain, Baoji, China | MK843818 |
|  | *T. ranunculoides* | Yunnan, China | MK253447 |
|  | *Calathodes oxycarpa* | Taibai Mountain, Shaanxi, China | MK569478 |
| Isopyreae | *Aquilegia coerulea* | Taibai Mountain, Shaanxi, China | MK569474 |
|  | *Urophysa rockii* | Mianyang, Sichuang, Chin | MK569502 |
|  | *Thalictrum minus* | Institute of Botany, CAS, Beijing, Chin | MK569500 |
|  | *Thalictrum thaloides* | China | MH092834 |
|  | *Dichocarpum fargesii* | Taibai Mountain, Shaanxi, China | MK569485 |
| Caltheae | *Caltha palustris* | Taibai Mountain, Shaanxi, China | MK569480 |
|  | *Stephania tetrandra* | China | MT859132 |
| Callianthemeae | *Callianthemum alatavicum* | Taibai Mountain, Shaanxi, China | MK253466 |
| Delphinieae | *Aconitum kusnezoffi* | Institute of Botany, CAS, Beijing, China | MK569468 |

UBU: Herbarium of National University of Mongolia; BJFC: Herbarium of Beijing Forestry University; EBL: Evolution Biology Laboratory of Northwest University; CAS: Chinese Academy of Science.

**Supplementary Table S4** Basic plastomes information of Adonideae species.

| Species | | *Calathodes oxycarpa* | *Megaleranthis saniculifolia* | *Trollius macropetalus* | *T. chinensis* | *T. farreri* | *T. ranunculoides* |
| --- | --- | --- | --- | --- | --- | --- | --- |
| Nucleotide length (bp) | Total | 160415 | 159924 | 16094 | 160191 | 160611 | 159666 |
|  | LCS | 89017 | 88326 | 88555 | 88522 | 88944 | 88134 |
|  | IR | 26497 | 26608 | 26624 | 26632 | 26568 | 26500 |
|  | SSC | 18404 | 18382 | 18291 | 18405 | 18532 | 11472 |
| GC content | Total | 38 | 38 | 38 | 38.1 | 38 | 38 |
|  | LCS | 36.2 | 36.3 | 36.3 | 36.3 | 36.3 | 36.3 |
|  | IR | 43.1 | 43 | 43 | 43.1 | 43.2 | 43.1 |
|  | SSC | 31.8 | 31.9 | 32 | 32 | 31.7 | 31.8 |
| Number of the gene | Total | 131 | 131 | 134 | 131 | 131 | 131 |
|  | CDS | 80 | 80 | 80 | 80 | 80 | 80 |
|  | tRNA | 30 | 30 | 30 | 30 | 30 | 30 |
|  | rRNA | 4 | 4 | 4 | 4 | 4 | 4 |

**Supplementary Table S5** Intron-containing genes in *Adonis mongolica*, *A. amurensis*, *A. pseudoamurensis*, *A. sutchenesis*, and *A. coerulea* plastomes

|  | Gene | Region | Exon I | Intron I | Exon II | Intron II | Exon III |
| --- | --- | --- | --- | --- | --- | --- | --- |
| 1 | *trn*K-UUU | LSC | 37 | 2518 | 35 |  |  |
| 2 | *rps*16 | LSC | 41, 41, 41, 41, 40 | 940 | 229, 229, 229, 229, 230 |  |  |
| 3 | *trn*G-UCC | LSC | 23 | 736 | 48 |  |  |
| 4 | *atp*F | LSC | 410, 411 | 772 | 145, 144 |  |  |
| 5 | *rpo*C1 | LSC | 432 | 787 | 1605 |  |  |
| 6 | *ycf*3 | LSC | 124 | 765 | 228 | 717 | 159 |
| 7 | *trn*L-UAA | LSC | 35 | 588 | 50 |  |  |
| 8 | *trn*V-UAC | LSC | 39 | 610 | 35 |  |  |
| 9 | *clp*P | LSC | 71, 70, 70, 71, 71 | 693 | 292, 290, 290, 292, 289 | 958 | 246 |
| 10 | *pet*B | LSC | 6, 6, 6, 6, 5 | 924 | 649, 650 |  |  |
| 11 | *pet*D | LSC | 8, 8, 8, 8, 7 | 762, 741 | 475, 496, 496, 496, 497 |  |  |
| 12 | *rpl*16 | LSC | 9 | 1205 | 399 |  |  |
| 13 | *rpl*2 | IR | 385 | 671 | 434 |  |  |
| 14 | *ndh*B | IR | 777 | 732 | 756 |  |  |
| 15 | *rps*12 | IR | (114) |  | 26 | 543 | 232 |
| 16 | *trn*I-GAU | IR | 37 | 949 | 35 |  |  |
| 17 | *trn*A-UGC | IR | 38 | 799 | 35 |  |  |
| 18 | *ndh*A | SSC | 550, 553, 552, 550, 550 | 1027 | 549, 546, 546, 546, 546 |  |  |

**Supplementary Table S6** Intron-containing genes in *T. macropetalus*, *T. chinensis*, *T. farreri*, *T. ranunculoides, C. oxycarpa*, and *M. saniculifolia* plastomes

|  | Gene | Region | Exon I | Intron I | Exon II | Intron II | Exon III |
| --- | --- | --- | --- | --- | --- | --- | --- |
| 1 | *trn*K-UUU | LSC | 37 | 2518 | 35 |  |  |
| 2 | *rps*16 | LSC | 41, 41, 41, 41, 40 | 940 | 229, 229, 229, 229, 230 |  |  |
| 3 | *trn*G-UCC | LSC | 23 | 736 | 48 |  |  |
| 4 | *atp*F | LSC | 145 | 738 | 410 |  |  |
| 5 | *rpo*C1 | LSC | 432 | 787 | 1605 |  |  |
| 6 | *ycf*3 | LSC | 124 | 765 | 228 | 717 | 159 |
| 7 | *trn*L-UAA | LSC | 35 | 588 | 50 |  |  |
| 8 | *trn*V-UAC | LSC | 39 | 610 | 35 |  |  |
| 9 | *clp*P | LSC | 71, 70, 70, 71, 71 | 693 | 292, 290, 290, 292, 289 | 958 | 246 |
| 10 | *pet*B | LSC | 6, 6, 6, 6, 5 | 924 | 649, 650 |  |  |
| 11 | *pet*D | LSC | 8, 8, 8, 8, 7 | 762, 741 | 475, 496, 496, 496, 497 |  |  |
| 12 | *rpl*16 | LSC | 9 | 1205 | 399 |  |  |
| 13 | *rpl*2 | LSC | 385 | 671 | 434 |  |  |
| 14 | *ndh*B | IR | 777 | 732 | 756 |  |  |
| 15 | *rps*12 | IR | (114) |  | 26 | 543 | 232 |
| 16 | *trn*I-GAU | IR | 37 | 949 | 35 |  |  |
| 17 | *trn*A-UGC | IR | 38 | 799 | 35 |  |  |
| 18 | *ndh*A | SSC | 550, 553, 552, 550, 550 | 1027 | 549, 546, 546, 546, 546 |  |  |


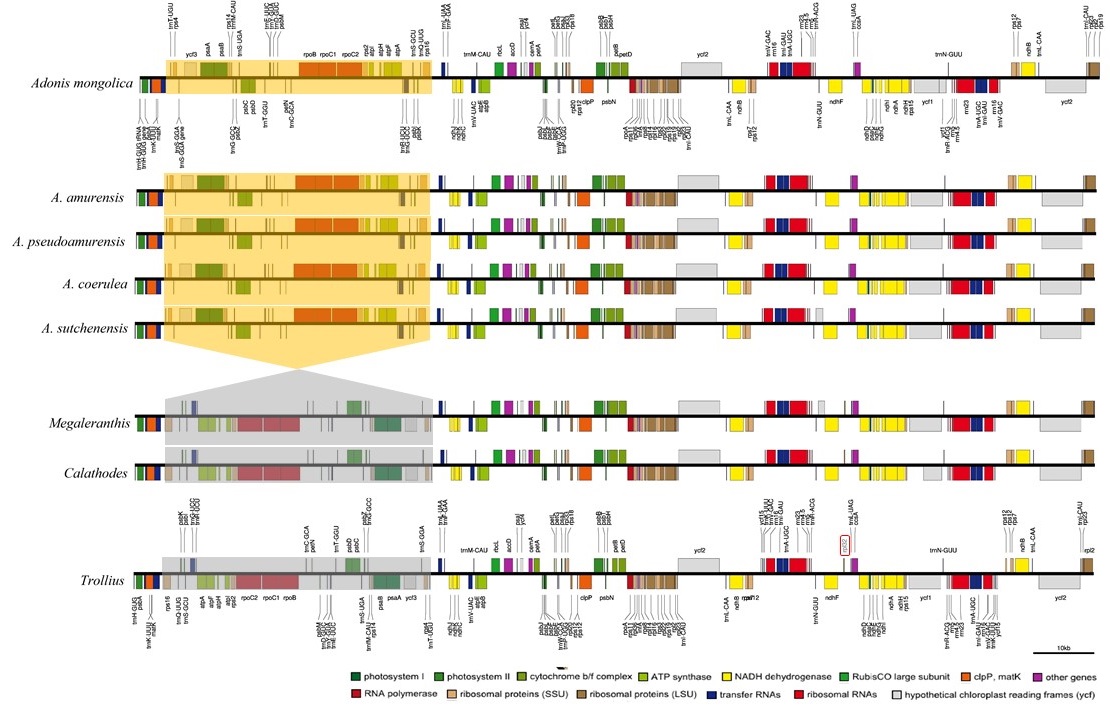


Supplementary Figure S1. Linear gene maps of plastomes of five *Adonis*, it compared with *Megaleranthis*, *Calathodes*, and *Trollius* genus within the Adonideae tribe. Genes are transcribed from left to right. Genes above the line are positioned in the forward direction (left to right). The inversion of genes of Adonis is represented with a light-yellow box. The origin of these inversion of the Adonideae is represented with a light-gray box. The *rpl*32 gene (marked with a red border) was completely lost from the *Adonis* plastomes.


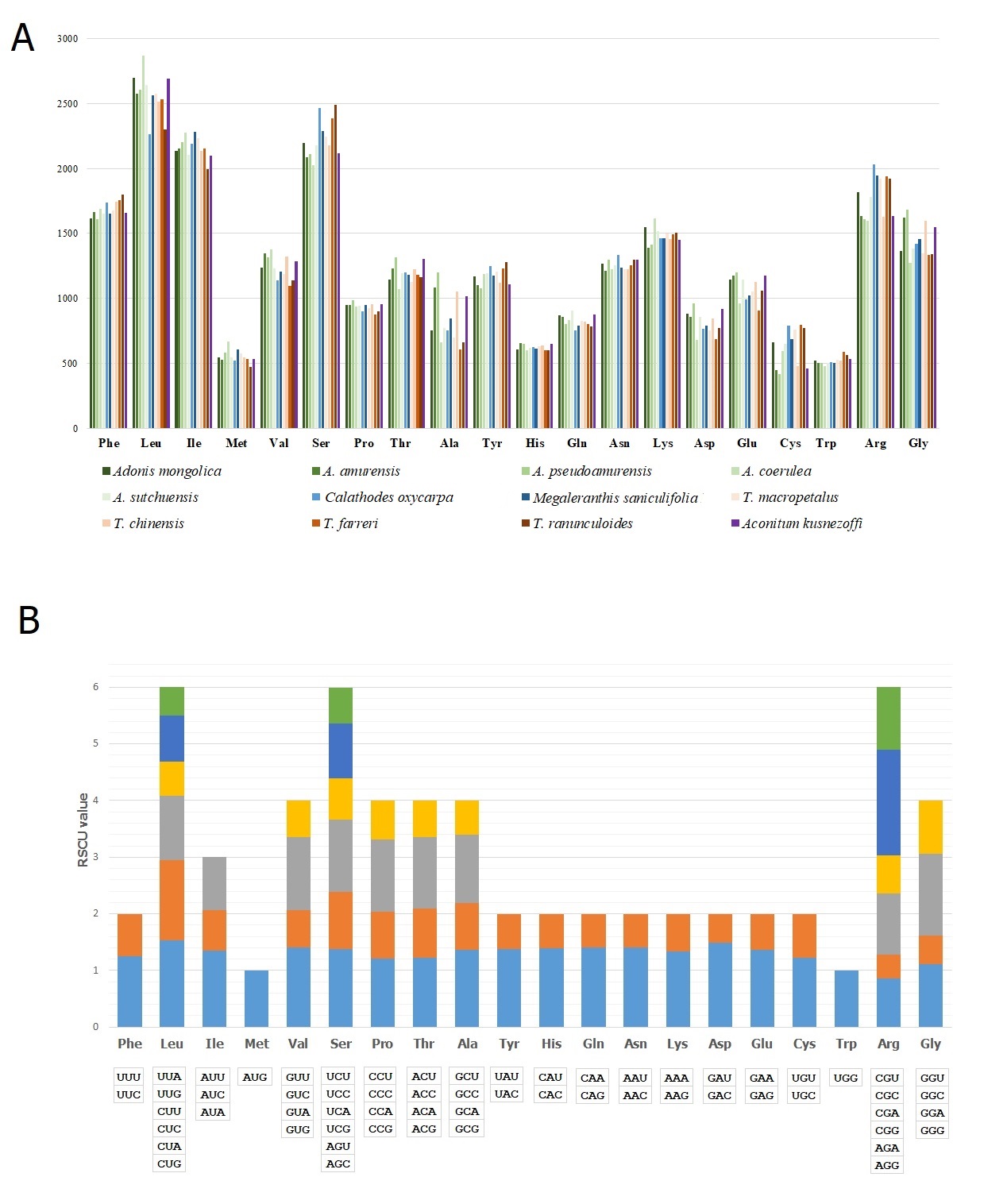


Supplementary Figure S2. Codon usage and anticodon recognition patterns of tribe Adonideae plastomes. (**a**) Amino acid frequencies for protein-coding sequences in five *Adonis* and four *Trollius*, one *Megaleranthis*, and one *Aconitum*. (**b**) Codon usage for 20 amino acids in protein-coding genes of the *Adonis* genus.


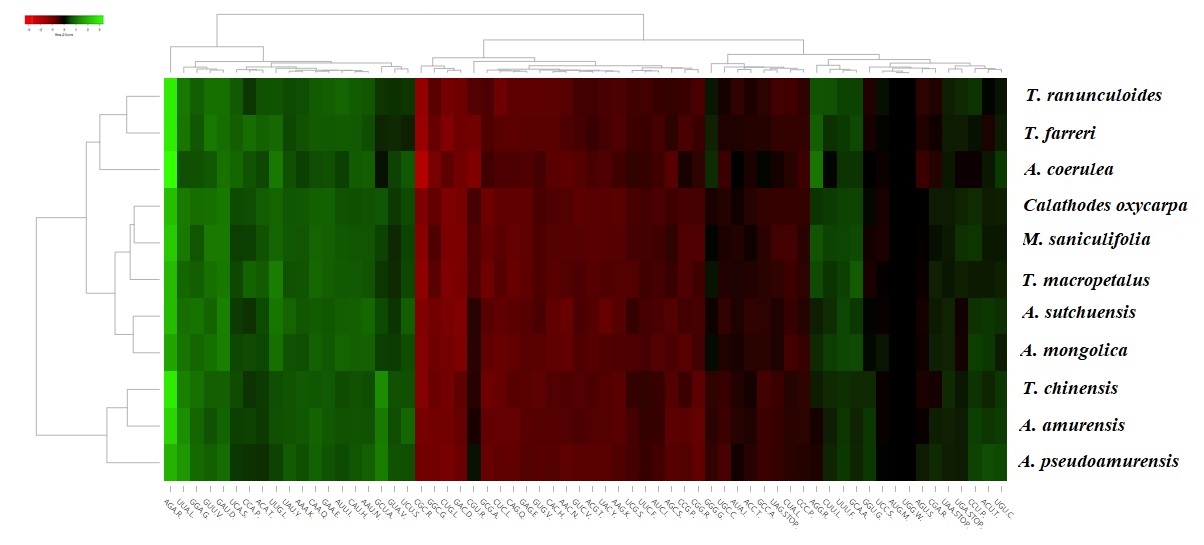


Supplementary Figure S3. Codon distribution of protein-coding genes in Adonideae plastomes. Green indicates a high relative synonymous codon usage (RSCU) value and red indicates a low RSCU value. Hierarchical clustering (average linkage method) was performed based on the codon patterns (x-axis).


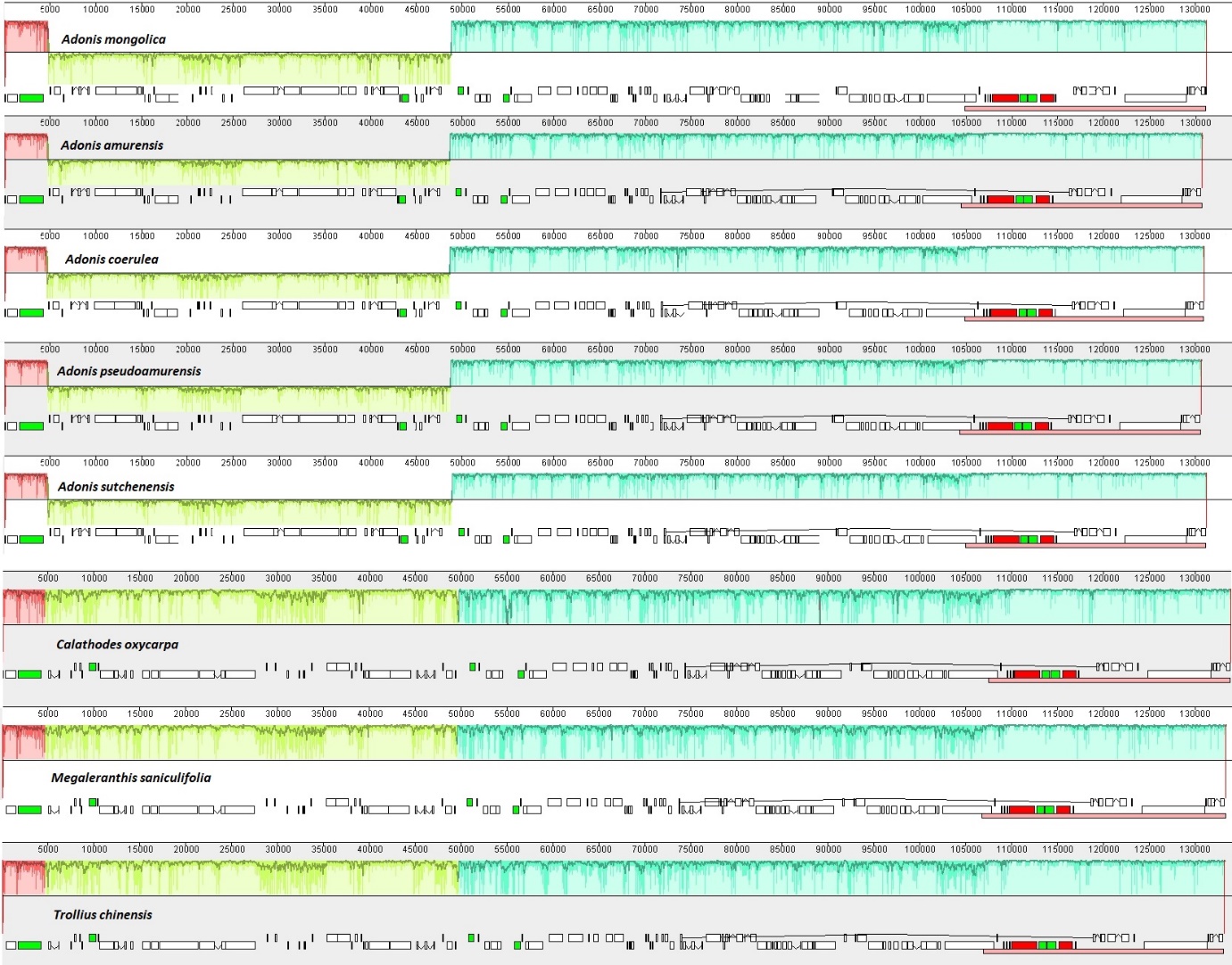
Supplementary Figure S4. MAUVE alignment of genera within Adonideae plastomes. Blocks on the top row are in the same orientation, while blocks on the bottom row are in inverse orientation. Boxes under each genome map represent protein-coding genes (white), rRNAs (red), and tRNAs (green).


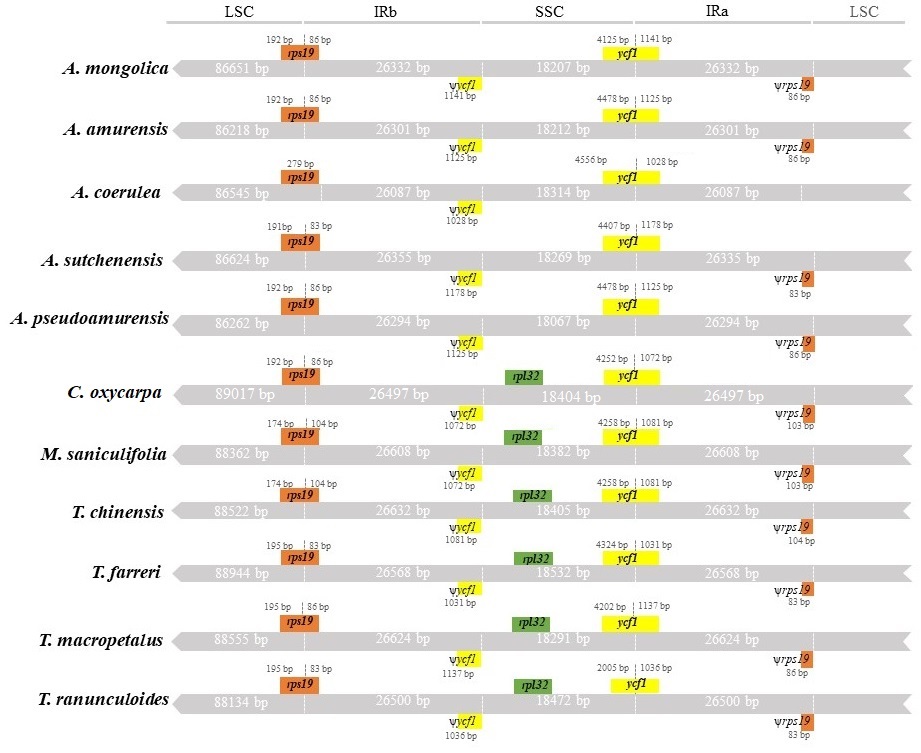


Supplementary Figure S5. Comparison of LSC, IR, and SSC junction positions among Adonideae plastomes. LSC, large single-copy region; SSC, small single-copy region; IR, inverted repeat.


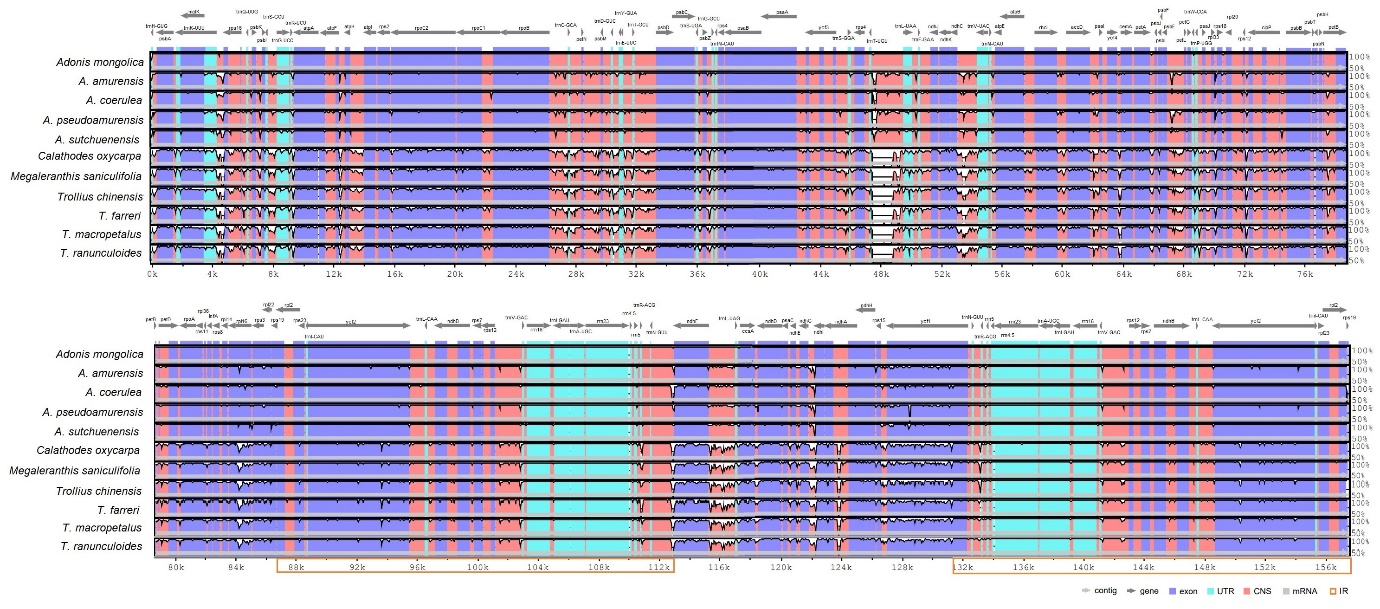


Supplementary Figure S6. Comparison of complete plastomes from eleven Adonideae species with *A. mongolica* as a reference using the mVISTA software. The gray arrows above the alignment indicate genes. Different colors represent different regions (coding and non-coding). The horizontal axis indicates the coordinates within the plastomes. The vertical scale represents the percentage of identity, ranging from 50 to 100%.


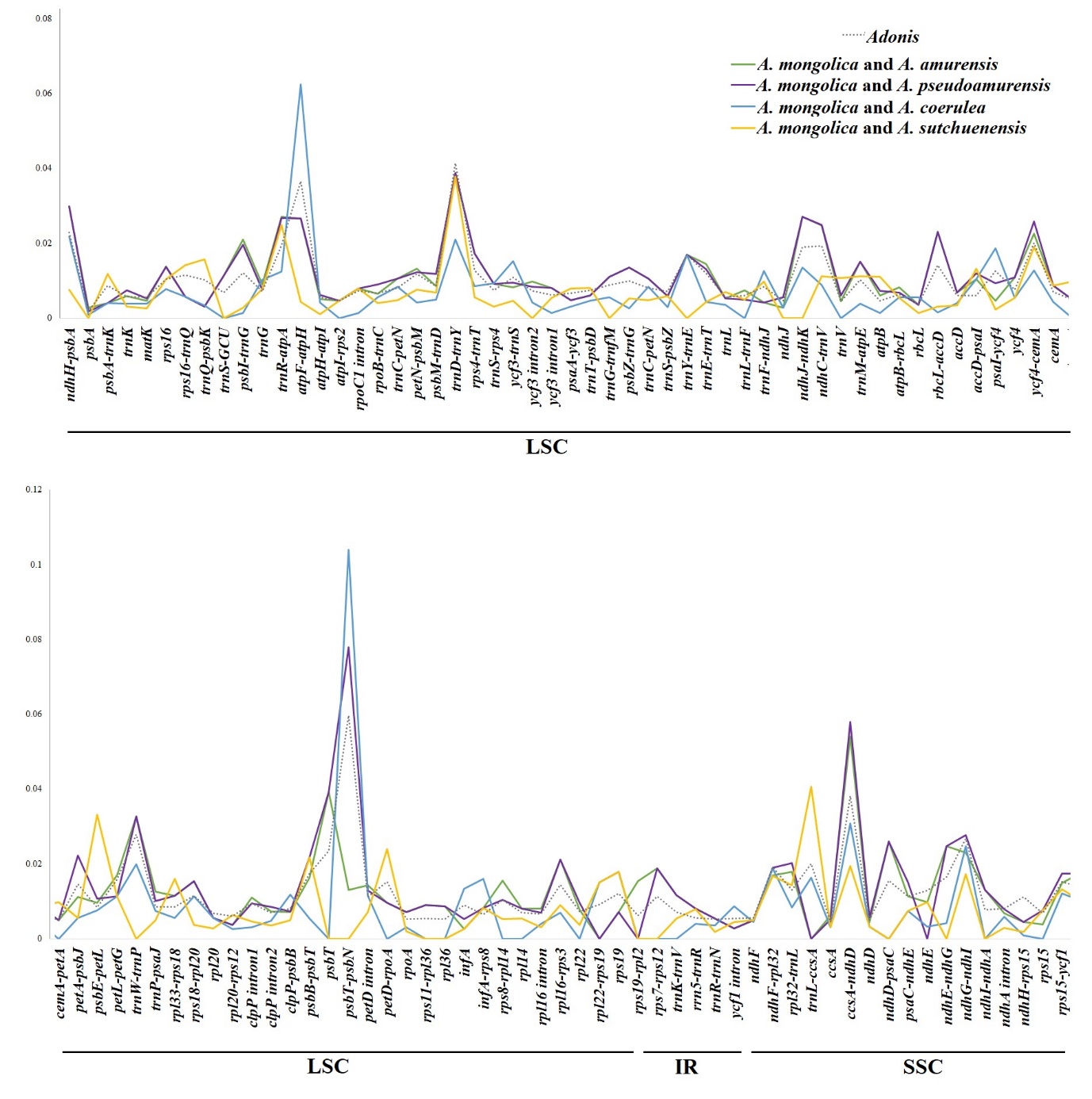


Supplementary Figure S7. Comparison of the nucleotide diversity (Pi) values among the *Adonis* species. The mean Pi value of five *Adonis* indicated by grey line.


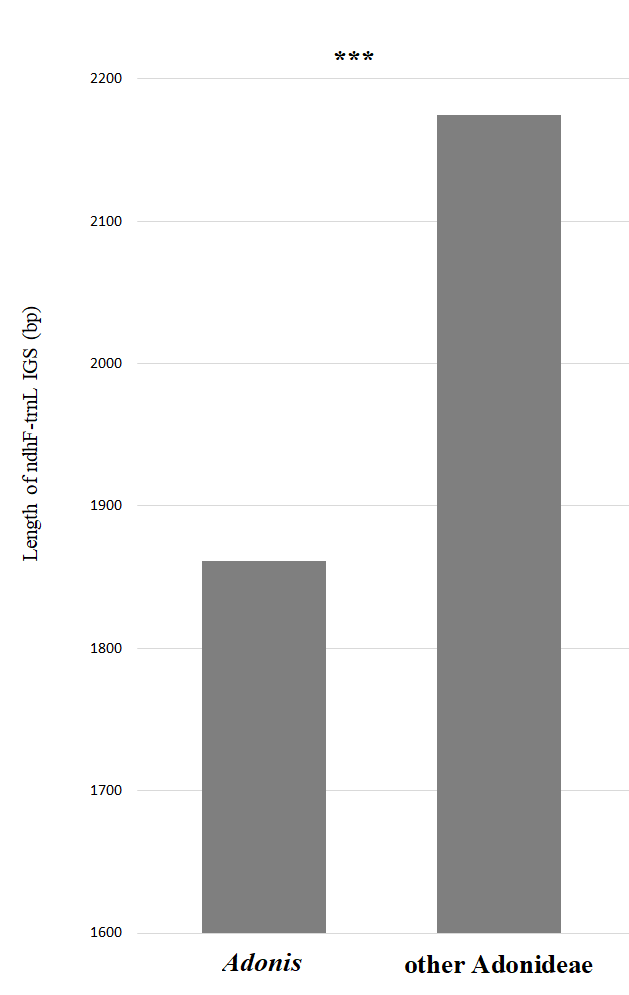


Supplementary Figure S8. The length variation of the *ndh*F-*trn*L intergenic spacer (IGS) including the *rpl*32 gene, which is absent in the genus *Adonis*. The differences of IGS length is marked as significant with an asterisk based on t-test (0 < *** < 0.001 < ** < 0.01 < * < 0.05).


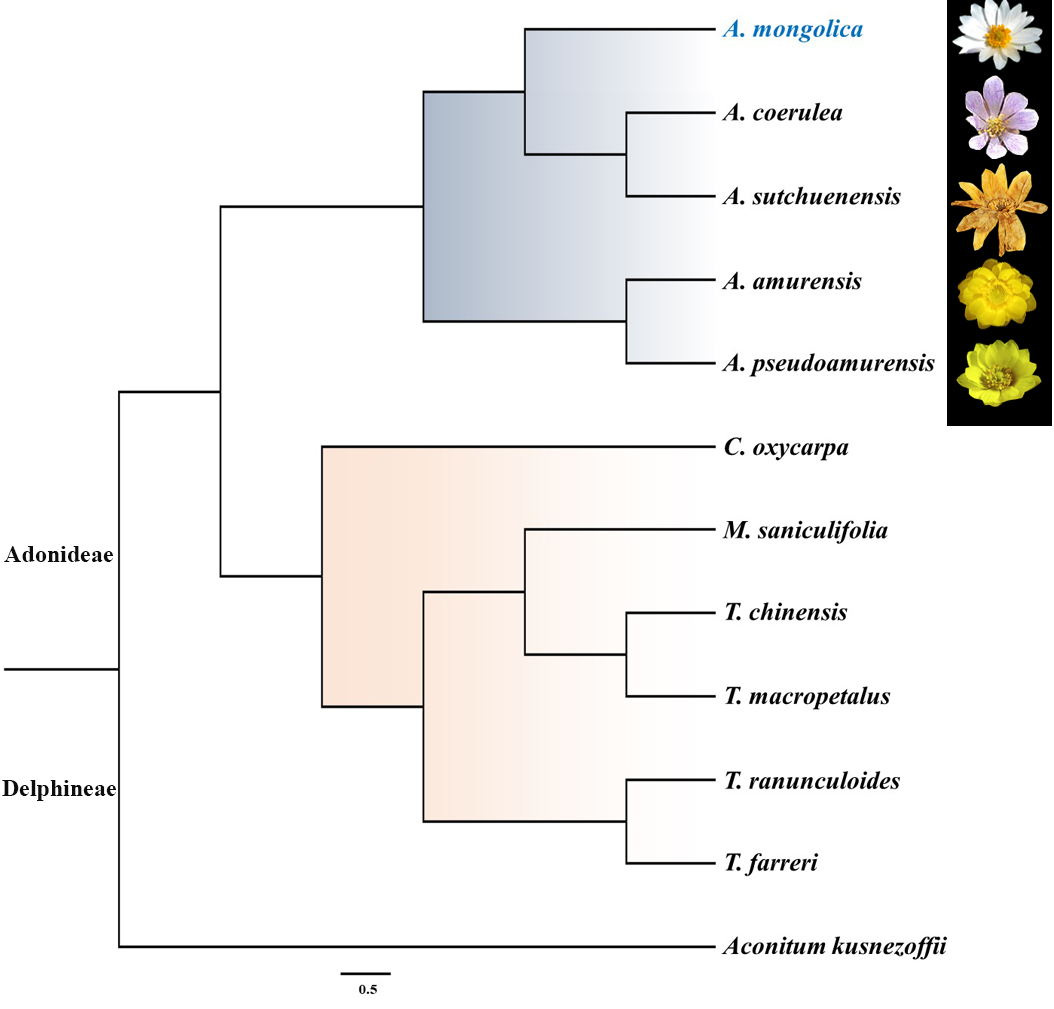


Supplementary Figure S9. Phylogenetic tree of the Adonideae species based on the protein-coding genes using Maximum Parsimony (MP), Bayesian Inference (BI), and Maximum Likelihood (ML). The MP topology is indicated with BI probabilities and ML bootstraps at each branch; maximum support values are not indicated (MP 100, PP 1, and ML 100). The plastome completed in this study is indicated in blue font. Each *Adonis* species is represented with a mature flower photograph.
